# Differences in Facial Expressions between Spontaneous and Posed Smiles: Automated Method by Action Units and Three-Dimensional Facial Landmarks

**DOI:** 10.3390/s20041199

**Published:** 2020-02-21

**Authors:** Seho Park, Kunyoung Lee, Jae-A Lim, Hyunwoong Ko, Taehoon Kim, Jung-In Lee, Hakrim Kim, Seong-Jae Han, Jeong-Shim Kim, Soowon Park, Jun-Young Lee, Eui Chul Lee

**Affiliations:** 1 Interdisciplinary Program in Cognitive Science, Seoul National University, Seoul 08826, Korea; parkho@snu.ac.kr (S.P.); powerzines@snu.ac.kr (H.K.); 2 Dental Research Institute, Seoul National University, School of Dentistry, Seoul 08826, Korea; 3 Department of Psychiatry, Seoul National University College of Medicine & SMG-SNU Boramae Medical Center, Seoul 03080, Korea; 4 Department of Computer Science, Sangmyung University, Seoul 03016, Korea; guy9284@gmail.com; 5 Seoul National University College of Medicine, Seoul 03080, Korea; taehoon.kim@snu.ac.kr (T.K.); pudu@naver.com (H.K.); haha940120@snu.ac.kr (J.-I.L.);; 6 Department of Education, Sejong University, Seoul 05006, Korea; swpark@emocog.com; 7 Department of Human Centered Artificial Intelligence, Sangmyung University, Seoul 03016, Korea

**Keywords:** emotion, facial recognition, facial asymmetry, three-dimensional analysis

## Abstract

Research on emotion recognition from facial expressions has found evidence of different muscle movements between genuine and posed smiles. To further confirm discrete movement intensities of each facial segment, we explored differences in facial expressions between spontaneous and posed smiles with three-dimensional facial landmarks. Advanced machine analysis was adopted to measure changes in the dynamics of 68 segmented facial regions. A total of 57 normal adults (19 men, 38 women) who displayed adequate posed and spontaneous facial expressions for happiness were included in the analyses. The results indicate that spontaneous smiles have higher intensities for upper face than lower face. On the other hand, posed smiles showed higher intensities in the lower part of the face. Furthermore, the 3D facial landmark technique revealed that the left eyebrow displayed stronger intensity during spontaneous smiles than the right eyebrow. These findings suggest a potential application of landmark based emotion recognition that spontaneous smiles can be distinguished from posed smiles via measuring relative intensities between the upper and lower face with a focus on left-sided asymmetry in the upper region.

## 1. Introduction

The smile plays a pivotal role in the exchange of emotional states in non-verbal communication [1]. Generally recognized as an epitome of positive affect, research on facial expression of happiness has long been considered as a major focus of emotional expression. The most obtrusive facial movements of a typical smile are known to engage the *zygomatic major*; a facial muscle on the cheek that is capable of pulling the corners of the mouth. Despite its ease of identification, smile is one of the complex expressions due to its varying origin of internal state [2]. Facial features of smile are regularly observed without positive feelings during communication. For instance, fake smiles may appear to obscure one’s true emotional state. Previous research has distinguished Duchenne smile as opposed to a fake smile with additional movements around the eyes, the *orbicularis oculi* region [3]. The appearance of Duchenne markers has been widely adopted as genuine, spontaneous smiles while the absence indicated posed smiles. [4,5].

Assessment of emotional state via facial expression is not limited to interpersonal communication. The demand for an accurate evaluation of human emotion has seen an increased significance with commercial applications in industries such as automotive and entertainment [6,7]. Unlike other devices that require direct contact, emotion recognition with facial expression can be administered with a camera, a readily available sensor [8]. Moreover, facial expression, as a non-invasive biomarker, has potential use in clinical application to detect neuropsychological disorders such as depression [9,10], schizophrenia [11], and Parkinson’s Disease [12]. Although bare human eyes are apt to recognize facial expressions to a certain degree, computerized algorithms have explored its advantages in the field. In recent years, there have been significant advances in machine analysis to analyze changes in dynamics of facial expressions [13,14,15]. Such movements can be analyzed with automated facial behavioral analysis tools such as TAUD [16], Affdex [17], and OKAO [18]. OpenFace is an open source toolkit to analyze facial movements based on Convolutional Neural Network algorithm. It is capable of detecting facial landmarks, gaze, head pose, and facial expression based on facial action units (AUs) according to the facial action coding system (FACS) [19,20,21,22].

Although AUs are reliable indicators of facial muscle contraction, previous studies have investigated that asymmetries in facial movement can also be an indicator of distinct emotional states. Facial asymmetry refers to relative differences in the expression intensity of either left or right side of the face that arises from hemispheric lateralization [2]. In general, the right cerebral hemisphere is known to be more involved with emotional expression than the left cerebral hemisphere, which leads to relatively increased movement on the left facial expressions [1]. The distinct emotion processing mechanisms between spontaneous and posed expressions have been explored. However, studies facilitating asymmetry to distinguish posed and spontaneous emotions have been incongruous [23,24]. Moreover, being innervated from different regions of the brain, the motor control of the upper and lower facial muscles is also known to be independent during spontaneous expressions [25,26]. Therefore, further analysis of each region of the face during posed and spontaneous positive emotion must be investigated to observe both vertical asymmetries and horizontal discrepancies.

Most of the previous studies on automatic facial expression analysis were based on AUs, which has limited capacity in terms of hemispheric lateralization. Therefore, the aim of the current study was to establish an algorithm that is capable of detecting overall discrepancies in both vertical and horizontal axes in facial landmarks and to apply it to enhance the discriminating power between posed and spontaneous facial expressions. To address this purpose adequately, we focused on geometric operations based on 3D facial landmarks. The result of the analysis will be discussed in terms of anatomy and neuropsychology.

## 2. Materials and Methods

### 2.1. Participants

Participants were recruited from SMG-SNU Boramae Medical Center and Sejong University in Seoul, South Korea; a total of 115 adult participants were enrolled in the study. The inclusion criteria were as follows: (1) aged between 18 and 40, (2) normal vision, hearing and cognitive function, and (3) able to understand the overall experimental procedures. Glasses and hearing aids were allowed if needed, but the data used for the analysis excluded the data from the subject wearing glasses. All participants provided written informed consent before participating in the study. This study was conducted in accordance with the Declaration of Helsinki, and the protocol was approved by the Institutional Review Board of SMG-SNU Boramae Medical Center (IRB No.30-2017-63).

### 2.2. Posed and Spontaneous Facial Expression Task

**Figure 1:**
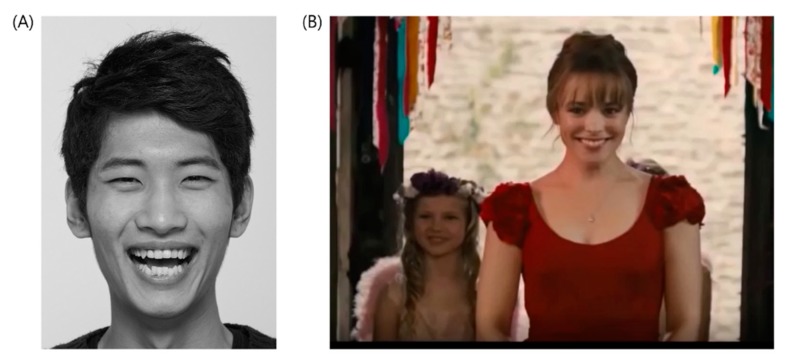
Stimuli used in posed and spontaneous facial emotion task. (**A**) Posed happy smiles. (**B**) Spontaneous happy smiles (film clip was taken from “About Time” movie clip).

Instructions for facial emotion task were given to the participants to draw posed and spontaneous facial expressions [27,28]. For example, a photograph of a person with a smiling face was presented; participants were asked to identify the emotion conveyed and “make a happy face for 15 s towards the camera” to be video recorded (posed emotion). Then, they watched a short film of approximately 120 s in duration to elicit positive emotions while their faces were video recorded by the camera (spontaneous emotion). Facial expression in their neutral-emotional status was also collected to make corrections on their ordinary facial characteristics. Figure 1 shows the stimuli used in the task.

### 2.3. Data Acquisition

The video recordings of the participants’ facial expressions were administered with Canon EOS 70D DSLR camera with 50 mm prime lens, 720 p resolution, and 60 fps frame rate. The experiments were done in a separate room with normal lighting condition. A screen was placed in front of the participants, about 60 cm apart from the seating position. The camera was mounted on a fixed stand about 120–140 cm above the ground to adequately capture the participants from their chest and above to make sure the whole face was included even with moderate head movements. The posed smiles were recorded for 15 s after a clear instruction to imitate a previously recognized happy face. The spontaneous facial expressions were video recorded for 60 s. The presence of smile was detected by OpenFace toolkit. Moreover, the videos of the facial expression were manually cross checked and confirmed. The movement of AU06 (cheek raiser) and AU12 (lip corner puller) were extracted in two measures. The toolkit provides trained predictors capable of determining the presence of AUs and their intensity [21,22]. The video recorded data of the two smiles were fundamentally distinguished by the experiment design. The two smile videos were collected with 317 moments of spontaneous smiles and 185 moments of posed smiles. As a result of the data collection, 58 of the 115 subjects wearing glasses were excluded from the analysis. Occlusion problems caused by the glasses’ frame and specular reflections on the spectacle lens surface can interfere with facial landmark extraction. Thus, a total of 57 data were included in the final analysis. Facial behavior analysis data such as AU06, AU12, and facial landmark were extracted from the final data collected to analyze the difference between spontaneous and posed smiles.

### 2.4. Self-Reported Measures

#### 2.4.1. Beck Depression Inventory (BDI2)

The Korean version of BDI involves 21 questions to evaluate the severity of depression, with its score ranging from 0 to 63 [29,30]. A higher score indicates severe depressive symptoms; the cutoff score is 18 in the Korean version [31].

#### 2.4.2. Beck Anxiety Inventory (BAI)

The Korean version of BAI utilizes 21 questions to measure the severity of anxiety, with its score ranging from 0 to 63 [32,33]. A higher BAI score indicates severe anxiety symptoms with its cutoff score of 19 [34].

#### 2.4.3. Toronto Alexithymia Scale (TAS)

A twenty-item TAS was developed and validated to measure the severity of alexithymia. A score ranging from 20 to 100 [35,36], with its cutoff score at 61 was used for the Korean version [37]. TAS is comprised of three sub-scales: identifying feeling, difficulty describing feeling, and externally oriented thinking.

### 2.5. Data Analysis

#### 2.5.1. Facial Behavior Analysis

Both posed and spontaneous facial expressions were under a preliminary investigation to confirm the presence of a smile via OpenFace 2.0, an open source toolkit to analyze facial landmarks and AUs [22]. AU06 (i.e., cheek raiser) and 12 (i.e., lip corner puller), which represent happiness, were evaluated and compared to those of neutral facial expression [20,38].

The AU estimation model used a real-time AU detection and intensity estimation system provided by OpenFace 2.0 [39]. This model extracts features based on facial appearances and geometric shapes that are classified to estimate the AU intensity using machine learning model. Moreover, this model demonstrates performance improvements in AU classifications and estimates through a person-specific normalization technique using neutral facial expression as a dynamic approach [21]. We used the AU estimation model to calculate and compare the AU intensities of posed and spontaneous smiles.

**Figure 2:**
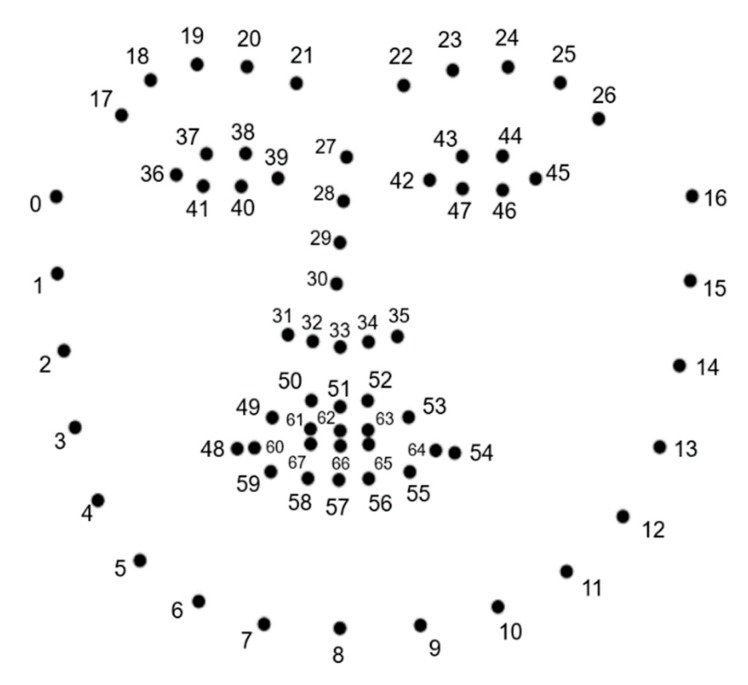
3D facial landmarks scatter plot and facial landmark’s index numbers.

The 3D facial landmark intensity estimation was measured by using each displacement between the landmark of the neutral expressions and the landmark of the smile expressions. The 68 point facial landmark annotations used in this study are represented in Figure 2. The 3D facial landmarks were extracted using convolutional experts constrained local model (CE-CLM) provided by OpenFace2.0 [40]. The performance of CE-CLM in OpenFace toolkit was measured on publicly available datasets such as 300-W [41], Menpo [42]. The size normalized median landmark error was used as the evaluation metric(ε), as shown in Equation (1).

(1) $$\varepsilon =\;\frac{1}{L}\;\underset{X\epsilon L}{\sum }\frac{d\left(\tilde{x},x\right)}{d_{scale}}$$

In the Equation (1), L is the number of facial landmarks, $d(\tilde{x},\;x)$ is the Euclidean distance between a predicted landmark and ground-truth landmark at the same index. $d_{scale}$ is a variable to normalize the difference caused by size variation and generally uses the distance between the two eyes. Performance evaluation results of CE-CLM in public dataset are shown in Table 1 [40].

**Figure 3:**
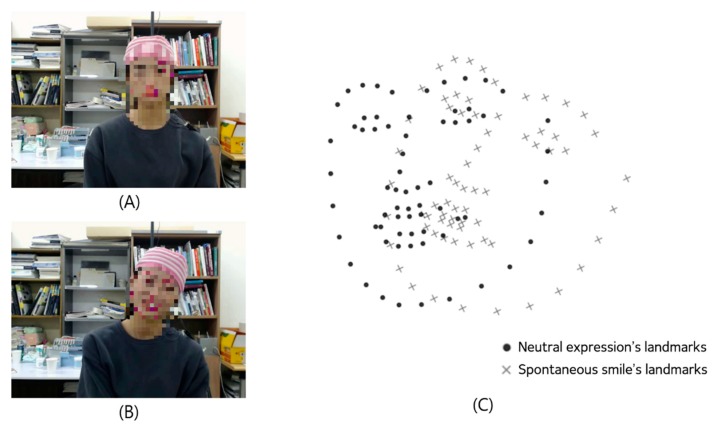
The example of alignment problem between facial landmarks in neutral and smile expression: (**A**) head position and angle during neutral expression, (**B**) head position and angle during smile expression, (**C**) example of misalignment problem between two facial landmarks.

**Figure 4:**
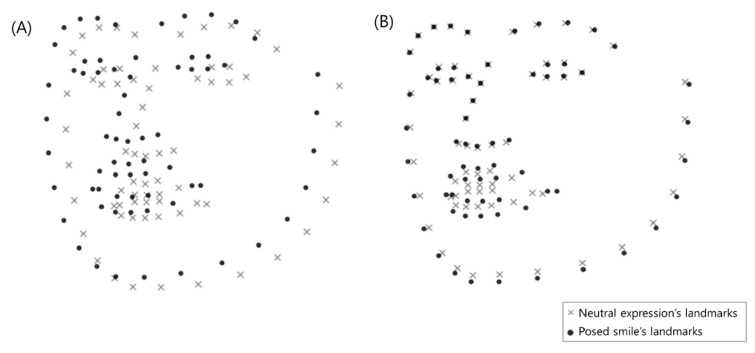
The example of aligning the two expression’s landmark sets: (**A**) scatter plot of two landmark data sets before aligning, (**B**) scatter plot of two landmark sets after aligning.

For the 3D facial landmark intensity estimation, we propose a new landmark-based displacement estimation method that resolves issues with incorrect displacement measurements. The proposed method has a contribution that it is capable of revising incorrect displacements caused by person-specific appearances, head movements, and rotations. Person-specific appearance problems occur because different people have different sizes and shapes of eyes, noses, and mouths, while some people have grimaces or smiles, even in their neutral state. In this study, the person-specific appearance problem is solved by measuring the displacement of smile expression landmark using the landmark of neutral expression as a basis. Moreover, as shown in Figure 3, when measuring facial landmarks of neutral expressions, posed and spontaneous smiles, the head position, and orientation of each expression were different from one another. Therefore, the 3D landmarks were required to be aligned to measure changes in each landmark. We used Sorkine et al.’s rigid motion computation to align the landmark sets in both expressions [43]. The aligning process of the two landmark sets is shown in Algorithm 1. The example of aligning the landmark sets of the expressions are shown in Figure 4. Through the processes above, we measured the changes of each landmark every frame and compared the facial movements of each area. As a result of aligning about 250,000 pairs of 57 subjects’ smiles and neutral expressions using rigid motion computation, the mean of residual error between each pair of fiducial points was 0.062 mm. These fiducial points were predefined landmarks for aligning landmarks of smiles and neutral expressions. In Figure 2, landmarks corresponding to the 27, 28, 29, 30, 39, and 42 indexes were selected as fiducial points.
**Algorithm 1.** Left and Right Facial Movement Measurement Algorithm.**Input:** Two 3D landmark sets of neutral expression and one of the spontaneous and posed smiles **Output:** Distance of landmarks between the two expressions * Facial landmark points were measured with reference to camera coordinate system. 1. Compute the weighted centroids of fiducial point sets $(p_{i},\;\;q_{i})\;$of the two landmark datasets * Fiducial point sets $(p_{i},\;\;q_{i})\;$are predefined landmarks for rigid body transformation. In Figure 2, landmarks corresponding to the 27, 28, 29, 30, 39, and 42 indexes were selected as fiducial points. $$\bar{p}=\;\frac{\sum_{i=1}^{n}w_{i}p_{i}}{\sum_{i=1}^{n}w_{i}}\;,\;\bar{\;q}=\;\frac{\sum_{i=1}^{n}w_{i}q_{i}}{\sum_{i=1}^{n}w_{i}},\;\;\;i=1,\;2,\;\ldots ,\;n.$$
2. Compute the centered vectors $x_{i\;}∶=\;p_{i}-\;\bar{p}\;,\;y_{i\;}∶=\;q_{i}-\;\bar{q}\;,\;\;i=1,\;2,\;\ldots ,\;n.$3. Compute covariance matrix $$\begin{array}{c} C=\mathrm{XW}Y^{T},\;X=[x_{1\;},x_{2\;},\ldots ,x_{n}]\;,\;Y=[y_{1\;},y_{2\;},\ldots ,y_{n}]\\ \mathrm{were}\;W\;\mathrm{is}\;\mathrm{diagonal}\;\mathrm{matrix}\;\mathrm{with}\;\mathrm{the}\;\mathrm{weight}\;w_{i} \end{array}$$
4. Compute the singular value decomposition $C=U\sum V^{T}$5. Compute rotation matrix $$R=V\left(\begin{array}{cc} \begin{array}{cc} 1 & \\ & 1 \end{array} & \\ & \begin{array}{cc} \ddots & \\ & \det\left(VU^{T}\right) \end{array} \end{array}\right)U^{T}$$6. Compute the optimal translation as $t=\;\bar{q}-R\bar{p}\;$7. Align the two 3D landmark sets using rigid body transformation $$\begin{array}{c} \hat{P}=\left(s*R\right)\bar{p}+t,\;\;\;\;\;\;s=\;\;\frac{({\bar{p}}^{T}\bar{q)}}{n}\\ \mathrm{where}\;\hat{P}\;\mathrm{is}\;\mathrm{an}\;\mathrm{aligned}\;3D\;\mathrm{landmark}\;\mathrm{set}\;\mathrm{and}\;s\;\mathrm{is}\;\mathrm{scale}\;\mathrm{factor}. \end{array}$$8. Compute distance of landmarks between the two expressions $d_{i\;}∶=\;\hat{p_{i}}-\;q_{i}\;,\;\;i=1,\;2,\;\ldots ,\;68.$


#### 2.5.2. Behavioral Data Analysis

Descriptive statistics for demographic variables were calculated to mean score and standard deviation (SD). Additionally, we compared the difference in mean scores on posed and spontaneous smile intensities based on analysis of variance (ANOVA) procedures. Comparison of AUs (smile expression: posed vs. spontaneous) were analyzed with repeated-measures ANOVA, and 3D facial landmarks (smile expression: posed vs. spontaneous × side: left vs. right) were analyzed by two-way repeated-measures ANOVA. Pairwise post-hoc comparisons with Bonferroni correction were conducted to 3D facial landmarks. A p-value <0.05 was considered statistically significant. All statistical analyses were performed using R software.

## 3. Results

Participants’ demographic information is described in Table 2. Age, education years, and the clinical scale data are represented by sex (19 males, 33.33%). Both men and women were within normal range with mean clinical scale scores on depression, anxiety, and alexithymia.

**Figure 5:**
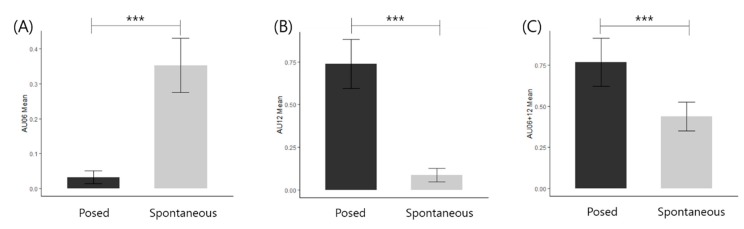
Contrast between posed and spontaneous smile intensities of AU. (**A**) AU06 (cheek raiser) mean score. (**B**) AU12 (lip corner puller) mean score. (**C**) AU06+AU12 mean score. (AU: Action Unit; *** P < 0.001.).

A repeated-measures ANOVA was computed with posed and spontaneous smile intensities according to AU06 (cheek raiser), AU12 (lip corner puller), and AU06+AU12 (see Table 3 and Figure 5). An AU06, located upper face, showed significantly higher intensities in spontaneous smiles than posed smiles. On the other hand, an AU12 corresponding to lower facial muscles had lower intensities in spontaneous smiles than posed smiles. The comparison of facial expression for the combination of AU06 and AU12 was identical to the pattern shown in AU12.

**Figure 6:**
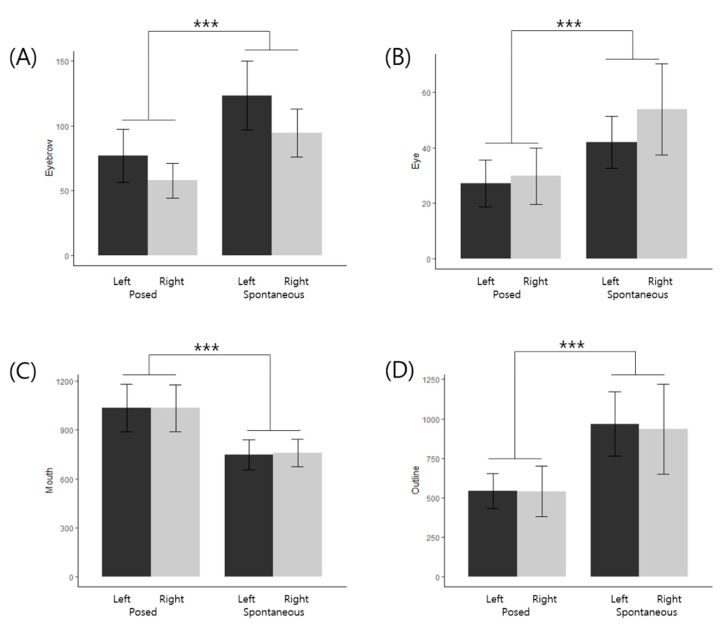
Classification of 3D landmarks in facial features to posed and spontaneous smiles with left and right faces. The difference in mean scores on posed/spontaneous smile intensities and left/right side of (**A**) Eyebrow. (**B**) Eye. (**C**) Mouth. (**D**) Outline. (*** P < 0.001).

**Figure 7:**
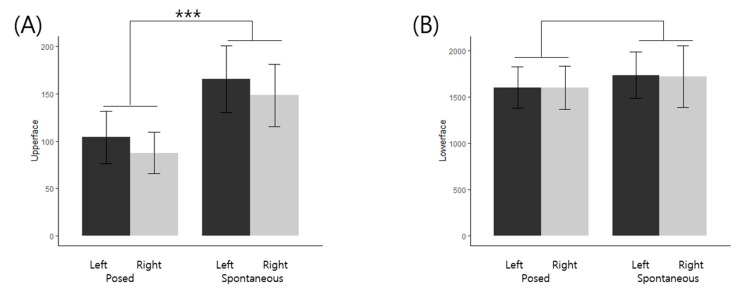
Classification of 3D landmarks in facial features to posed and spontaneous smiles with left and right faces. The difference in mean scores on posed/spontaneous smile intensities and left/right side of (**A**) Upper face. (**B**) Lower face. (*** P < 0.001).

With respect to the 3D facial landmarks, a repeated-measures ANOVA was performed to find out the differences in smile expression (posed vs. spontaneous) and side of face (left vs. right). There were significant main effects of smile expression in eyebrow, eye, mouth, outline, and upper face features. Pairwise t test with Bonferroni correction revealed that eyebrows were significantly higher intensity on left side (P < 0.001) and spontaneous expression (P < 0.001), and eyes had more intensities in spontaneous expression (P < 0.001). Both outline and upper face indicated more intense expression in spontaneous smiles (P_outline_ < 0.001; P_upper face_ < 0.001). Mouth, on the other hand, showed stronger intensity in posed expression (P < 0.001). The results are shown in Table 4, Figure 6 and Figure 7.

## 4. Discussion and Conclusions

### 4.1. AU Intensity Estimation

The facial movements of spontaneous and posed smiles have displayed distinctive patterns. The intensity of AU06 was significantly higher in spontaneous smile than that of posed smile, while the intensity of AU12 was significantly higher in posed smiles. In other words, the area around the eyes was more actively involved during a genuine smile. On the other hand, the area around the mouth was more active when the smile was voluntarily posed. These results are consistent with previous findings that expression of spontaneously occurring happiness or satisfaction can be distinguished from a fake posed smile by noticeable movements of the upper cheek and muscles around the eyes [44]. These behavioral differences between the two regions are the result of differences in neural innervation, where the facial nerve is specialized for communication [45]. Most people are capable of voluntary control of mouth regions where they are unilateral in nature and mostly managed by the frontal lobes. However, the deliberate motion of the eye regions is only possible for a limited number of people, which is about 20% [46]. Unlike the mouth and the lower part of the face, the upper regions are bilaterally controlled via the limbic system [47].

The overall intensity of spontaneous and posed smiles, which involves simultaneous activation of both *zygomaticus major* and *orbicularis oculi* (AU06+AU12), was higher in posed smiles than spontaneous smiles. This result may be due to the stimulus for the spontaneous condition; a wedding scene from a movie may not be able to arouse a significant degree of genuine emotional change for a wide open-mouth smile. In fact, the process of spontaneous smile is much more complicated than a voluntary act of posing a smiling face. Regulation of posed movement of the face is generated from the lower portion of the precentral gyrus and projects to the facial nucleus [1]. However, the anatomical foundation of spontaneous emotional expression involves more structures with complicated pathways than that of posed expression. Spontaneous expressions are known to arise from thalamus and/or the globus pallidus and project to the facial nucleus via several different routes [1,3]. Since it is difficult to estimate how the stimuli are perceived to arouse natural smile, trained and automated algorithm to incorporate large sample is an indispensable aspect of facial expression analysis.

### 4.2. 3D Facial Landmark Intensity Estimation

We proposed a person-specific normalization method for facial landmark analysis to detect changes of intensity in facial muscle movements. The algorithm in our method has shown proficiency in measuring partial differences in each regions of the face over both hemifaces. The result of our analysis indicates significant differences in intensities between the left and the right eyebrows. The movement of left eyebrows displayed greater intensity than that of right eyebrows in both posed and spontaneous smiles. Moreover, the overall intensities of eyebrows were higher in spontaneous smiles than posed smiles, and the differences were also significant. On the other hand, the movement of the eyes displayed a relatively larger right-biased intensity, which is an opposite trend from the eyebrows. The *zygomatic major*, which is a major muscle in the lower region, influences face areas under the eyebrows [2]. Therefore, it is possible to see different lateralization patterns from the eyebrows and the eyes. Such results were not observed with conventional AUs, which sums up the whole movement in the eye area. Moreover, the aligning algorithm may have enabled the detection of subtle differences in intensities.

Although there was no significant interaction between expression and facial asymmetry, our observation on the greater intensity of the left *orbicularis oculi* region is consistent with previous studies. According to the right hemisphere hypothesis, the right hemisphere dominates the mediation of emotion, and therefore, facial expressions should be more evident on the left side of the face in general [25]. Our results also indicated that the upper face displayed significantly more movement in spontaneous than posed condition, which could serve as a reasonable indicator of a genuine smile. Our result is consistent with previous research that facial movement discrepancies during spontaneous expressions are more evident between the upper and lower faces than along the vertical axis [25,26]. The findings support the Component theory of facial expressions that the upper and lower facial expressions are fundamentally distinct at the level of both behavior and emotion; the upper being innervated from both right and left medial cortical projections while the lower from lateral cortical projection [48].

### 4.3. Limitations and Future Directions

Although facilitating the landmark-based facial recognition system is sufficient for detecting differences between posed and spontaneous smiles, differently weighing each point may enable the system to uncover more latent facial features. The range of muscle movements varies greatly between each facial region. For instance, the landmarks around the nose have significantly less range of motion than that of mouth. Future studies can be conducted with enhanced discriminating power by incorporating the possible range of motion on each landmark point.

Another limitation to the current study pertains to a relative disparity between the training data and actual data from the participants. Ekman stated that different aspects of expression are both universal and culture specific [49]. The participants for the current study were all Koreans, while the training data used to detect FACS are from populations of different races and cultures. Moreover, the posed smile falls under the category of social-emotional response, which is learned through social contexts [26]. Therefore, unlike spontaneous smile, the posed smile is prone to discordance between the training data and the actual recordings of the participants.

As noted in the previous section, the authenticity of spontaneous smile is subject to individual differences. Repeated experiments with the same participants may significantly increase the authenticity of the data. Therefore, further research must consider follow-up experiments with previously enrolled participants for precise analysis and reliability of the current method. Future research may also consider additional participants with emotion regulation disabilities or the elderly population to explore areas of emotion expression and recognition in terms of healthy aging.

### 4.4. Conclusions

The results reported in the present study support the automated 3D facial landmark detection could effectively distinguish genuine smiles from posed smiles. According to our data, the upper face is more involved during spontaneous expression than the lower face. Specifically, the left eyebrow could serve as a key indicator of a positive emotional state. In addition, under the same circumstances, increased movement around the mouth with relatively lower intensity in the upper face may indicate the smile is posed. The horizontal asymmetry and vertical discrepancy have been proven to be useful measures of facial expression in positive emotion. Increased movement in the upper face may serve as a partial indicator of a genuine smile. The results of this study are obtained from normal adults and can be used as a basic methodology for analyzing and for identification of clinical features of facial expression data.

## Figures and Tables

**Table 1:** The size normalized median landmark error of CE-CLM on 300-W and Menpo [40,41,42].

Helen and LFPW (300-W)		iBUG (300-W)		Menpo (Frontal Face)	
with outline (68)	without outline (49)	with outline (68)	without outline (49)	with outline (68)	without outline (49)
0.00315	0.00230	0.00531	0.00386	0.00223	0.00174

* These error values are the size normalized values of the distance between annotated ground-truth landmark point and predicted landmark point.

**Table 2:** Characteristics of participants.

	Male (n = 19)	Female (n = 38)	Total (n = 57)
	mean ± SD ^1^	mean ± SD ^1^	mean ± SD ^1^
Age	22.68 ± 2.16	22.55 ± 4.32	22.60 ± 3.72
Education (year)	15.05 ± 1.39	14.66 ± 1.02	14.79 ± 1.16
BDI ^2^	8.53 ± 5.30	12.13 ± 7.77	10.93 ± 7.20
BAI ^3^	2.47 ± 3.08	5.24 ± 5.08	4.32 ± 4.67
TAS ^4^	43.68 ± 7.59	45.79 ± 9.23	45.09 ± 8.71

^1^ SD: Standard Deviation, ^2^ BDI: Beck Depression Inventory, ^3^ BAI: Beck Anxiety Inventory, ^4^ TAS: Toronto Alexithymia Scale.

**Table 3:** Comparative mean AU ^2^ according to different type of smile expression.

	Posed (n = 57)	Spontaneous (n = 57)	*F*	*p*
	mean ± SD ^1^	mean ± SD ^1^		
**AU06** **(cheek raiser)**	0.03 ± 0.07	0.35 ± 0.29	81.48	<0.001
**AU12** **(lip corner puller)**	0.74 ± 0.54	0.09 ± 0.15	96.74	<0.001
**AU06+12** **(both)**	0.77 ± 0.55	0.44 ± 0.33	18.80	<0.001

^1^ SD: Standard Deviation, ^2^ AU: Action Unit.

**Table 4:** Comparison of smile expression by left- and right face based on facial landmarks.

	Expression^1^	Left (n = 57)	Right (n = 57)	*Expression*	*Side*	Pairwise Comparison
		mean ± SD ^2^	mean ± SD ^2^			
Eyebrow	Posed	76.93 ± 76.23	57.78 ± 50.43	20.03***	18.26***	Spontaneous > Posed Left > Right
	Spontaneous	123.23 ± 100.15	94.45 ± 69.06			
Eye	Posed	27.13 ± 31.81	29.83 ± 38.17	13.28***	6.15**	Spontaneous > Posed Right > Left
	Spontaneous	42.03 ± 35.39	53.74 ± 61.97			
Nose	Posed	25.56 ± 13.03	26.94 ± 13.64	1.68	4.68	*Not significant*
	Spontaneous	22.63 ± 11.11	25.72 ± 14.18			
Mouth	Posed	1035.13 ± 547.91	1033.77 ± 546.84	24.13***	0.06	Posed > Spontaneous
	Spontaneous	746.59 ± 347.66	760.45 ± 321.07			
Outline	Posed	541.97 ± 415.72	540.11 ± 602.22	15.48***	0.10	Spontaneous > Posed
	Spontaneous	968.60 ± 768.52	935.30 ± 1070.48			
Upper face (Eyebrow+ Eye)	Posed	104.07 ± 104.90	87.60 ± 83.05	18.32***	8.96	Spontaneous > Posed
	Spontaneous	165.26 ± 132.66	148.19 ± 123.49			
Lower face (Nose+ Mouth+ Outline)	Posed	1602.65 ± 845.05	1600.82 ± 882.75	1.07	0.02	*Not significant*
	Spontaneous	1737.81 ± 953.92	1721.46 ± 1250.97			

^1^ Expression (Posed, Spontaneous), ^2^ SD: Standard Deviation.
